# Real-world patient journey and unmet needs of people with hidradenitis suppurativa: Social media research in China

**DOI:** 10.1016/j.jdin.2026.08.018

**Published:** 2026-09-17

**Authors:** John R. Ingram, Wen-Yu Wu, Wenbo Bu, Li Yi, Mei Go, Ivette Alarcon, Amit Garg

**Affiliations:** a Division of Infection and Immunity, Cardiff University, Cardiff, UK; b Department of Dermatology, Huashan Hospital Fudan University, Shanghai, China; c Hospital for Skin Diseases, Institute of Dermatology, Chinese Academy of Medical Sciences & Peking Union Medical College, Nanjing, China; d China Chronic Skin Disease Fund, Beijing Health Alliance Charitable Foundation, Beijing, China; e Novartis Pharma AG, Basel, Switzerland; f Northwell Health, New Hyde Park, New York

**Keywords:** China, disease burden, hidradenitis suppurativa, pain, patient journey, quality of life

*To the Editor:* Hidradenitis suppurativa (HS) is a chronic, inflammatory skin disease that substantially impairs patients’ quality of life (QoL).^1^ In 2023, HS was classified as a rare disease in China, enabling policy initiatives to enhance public awareness, diagnosis, and treatment access.^2^ Social media research (SMR) is increasingly used to gain insights into patient experiences and generate real-world data beyond clinical settings.^3^ The objective of this study was to understand key discussion topics, patient journey, symptoms, impact of HS on QoL, and unmet needs through assessment of social media posts in China. A post was categorized as containing QoL-related themes if it included clear indications of negative impact on daily life, such as:

• Physical discomfort (eg, pain, mobility limitations)
• Emotional or psychological distress (eg, frustration, embarrassment, anxiety)
• Disruption to daily activities, work, or social interactions, etc.

Duplicates were removed during data filtering. Each post was treated as an independent observation and served as the unit of analysis. This retrospective study (Sep 2022-Sep 2024) analyzed HS-related posts in Mandarin and English across social networking platforms and Online Diagnostics, focusing on HS-specific terms (Supplementary Fig 1 and Table I, available via Mendeley at https://data.mendeley.com/datasets/4xtf67dgm9/1). Detailed insights were generated against each metric (demographics, QoL impact, patient journey stages, key concerns, and unmet needs) to help answer the research questions (Supplementary Appendix, available via Mendeley at https://data.mendeley.com/datasets/4xtf67dgm9/1).

A total of 14,739 mentions of HS were captured across platforms, with WeChat (46%), Weibo (19%), and Xiaohongshu (18%) being most used. Of these, 1410 patient-centric posts were identified; 1016 relevant posts were coded for insights (Supplementary Fig 2, available via Mendeley at https://data.mendeley.com/datasets/4xtf67dgm9/1). Most posts were from people with HS, with males and females having a similar share of conversations where gender was identifiable.

The most frequently discussed topics included patient journey (*n* = 874), QoL impact (*n* = 559), and affected body parts (*n* = 385). QoL impact was identified in 559 posts (Fig 1); emotional challenges were most frequently reported (86%; 483/559) followed by physical (56%; 315/559) and functional challenges (11%; 62/559). Social issues (3%; 17/559) and financial concerns (1%; 8/559) were also mentioned. Posts describing consultations (*n* = 43) most commonly mentioned persistent pain (*n* = 21) and pus (*n* = 15).

**Fig 1:**
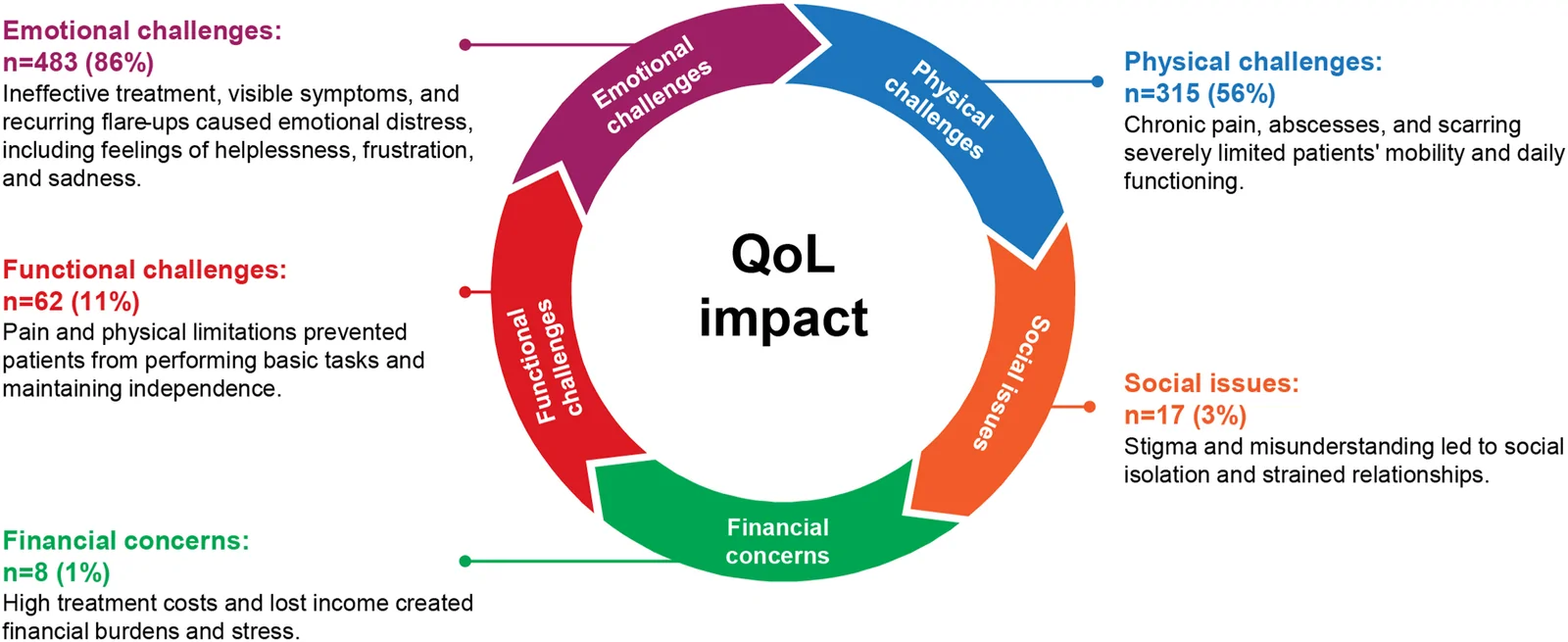
Impact on quality of life QoL, quality of life. The total may be more than 100% due to multiple themes mentioned in a post.

Among treatment-related posts (*n* = 694) surgery (*n* = 329) was most mentioned, followed by antibiotics (*n* = 133) and traditional Chinese medicine (*n* = 116). Most common reason for treatment discontinuation (*n* = 77) was inefficacy, followed by side effects. Drivers of current treatment were based on doctor’s recommendation and efficacy, while barriers included ineffective treatment and side effects. Lack of effective, sustained treatment was identified as a substantial unmet need (Fig 2). In China, no biologic therapies were approved for HS during the study period, which may have influenced the treatment patterns observed in the posts.

**Fig 2:**
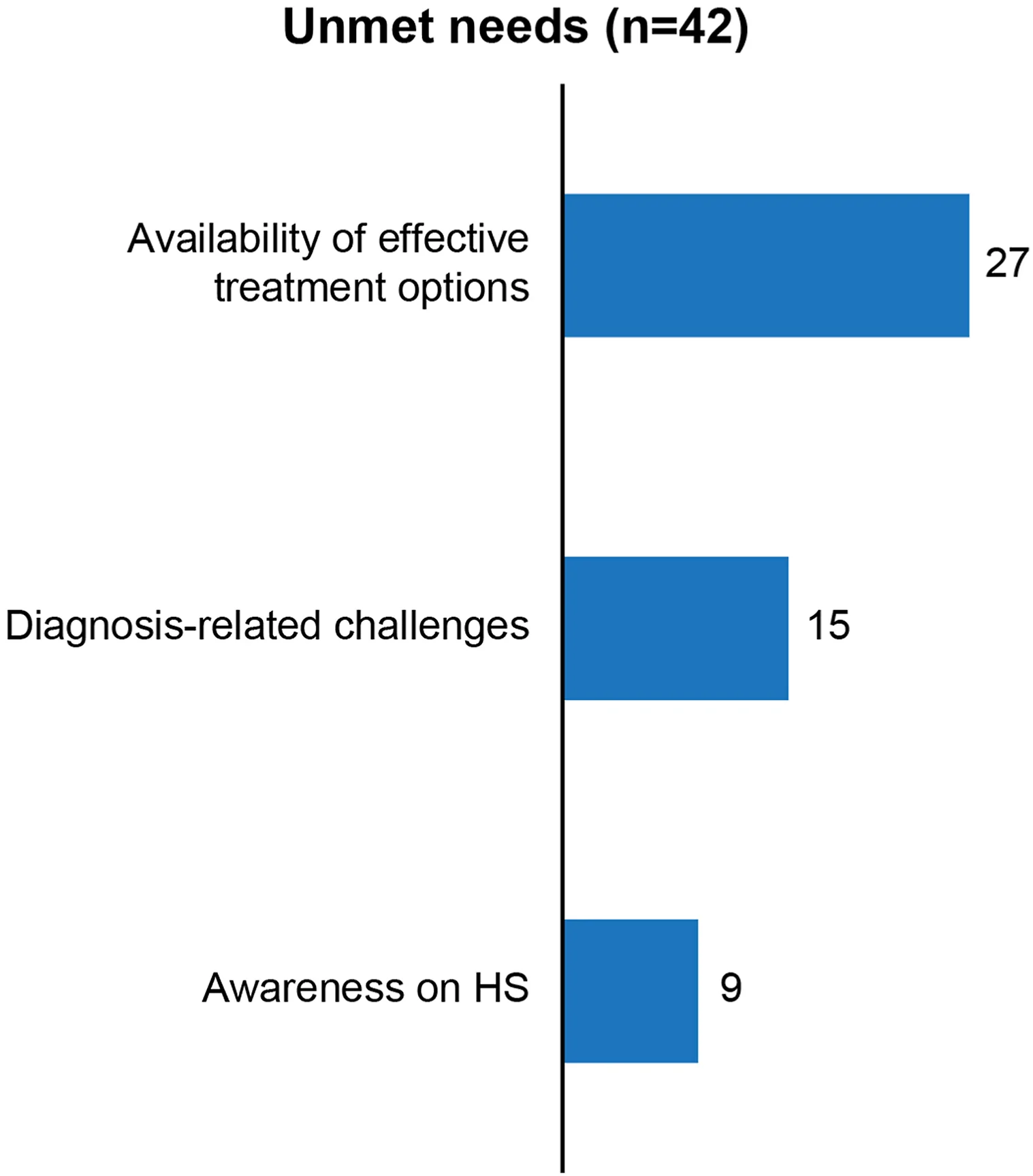
Unmet needs emphasized by HCPs HCP, healthcare professional; HS, hidradenitis suppurativa.

The results of this SMR analysis reveal frequent discussion of disease burden, QoL-related challenges, and unmet needs in HS-related social media posts from China. These data indicate a need for improved awareness, timely diagnosis, and access to effective and sustained treatments to reduce the substantial burden of living with HS in China, including most bothersome symptoms (pain/pus).

### Declaration of generative AI and AI-assisted technologies in the writing process

No AI tools were used in the preparation of this manuscript.

## Conflicts of interest

Ingram received stipend as an immediate past Editor-in-Chief of the British Journal of Dermatology and an authorship honorarium from UpToDate. He is a consultant for AbbVie, Bluefin Biomedicine, Boehringer Ingelheim, Cantargia, ChemoCentryx, Citryll, Elasmogen, Engitix, Incyte, Indero, InflaRx, Insmed, Kymera Therapeutics, MoonLake, Novartis, UCB Pharma, UNION Therapeutics, and Viela Bio. He is a co-copyright holder of HiSQOL, HIDE, Investigator Global Assessment and Patient Global Assessment instruments for HS, and his department receives income from copyright of the Dermatology Life Quality Index (DLQI) and related instruments. Wu received consulting fees from Novartis for his contributions to the development of research. Bu received consulting fees from Novartis for his contributions to the development of research. Yi has no conflict of interest to disclose. Go is an employee of Novartis. Alarcon is an employee of Novartis and holds company stock. Garg is an advisor for AbbVie, Almirall, Bluefin Biomedicine, Boehringer Ingelheim, Immunitas Therapeutics, Incyte, Insmed, Navigator Medicines, Novartis, Oruka Therapeutics, Otsuka Pharmaceutical, Pfizer, Priovant, Sun Pharma, Takeda, UCB, and Zura Bio.
